# Swine acute diarrhea syndrome coronavirus nucleocapsid protein antagonizes the IFN response through inhibiting TRIM25 oligomerization and functional activation of RIG-I/TRIM25

**DOI:** 10.1186/s13567-024-01303-z

**Published:** 2024-04-08

**Authors:** Jiyu Zhang, Hongyan Shi, Liaoyuan Zhang, Tingshuai Feng, Jianfei Chen, Xin Zhang, Zhaoyang Ji, Zhaoyang Jing, Xiaoyuan Zhu, Dakai Liu, Xiaoman Yang, Miaomiao Zeng, Da Shi, Li Feng

**Affiliations:** State Key Laboratory for Animal Disease Control and Prevention, Harbin Veterinary Research Institute, Chinese Academy of Agricultural Sciences, Xiangfang District, Haping Road 678, Harbin, 150069 China

**Keywords:** Swine acute diarrhea syndrome coronavirus, nucleocapsid, interferon, RIG-I, TRIM25

## Abstract

Swine acute diarrhea syndrome coronavirus (SADS-CoV), an emerging *Alpha-coronavirus*, brings huge economic loss in swine industry. Interferons (IFNs) participate in a frontline antiviral defense mechanism triggering the activation of numerous downstream antiviral genes. Here, we demonstrated that TRIM25 overexpression significantly inhibited SADS-CoV replication, whereas TRIM25 deficiency markedly increased viral yield. We found that SADS-CoV N protein suppressed interferon-beta (IFN-β) production induced by Sendai virus (SeV) or poly(I:C). Moreover, we determined that SADS-CoV N protein interacted with RIG-I N-terminal two caspase activation and recruitment domains (2CARDs) and TRIM25 coiled-coil dimerization (CCD) domain. The interaction of SADS-CoV N protein with RIG-I and TRIM25 caused TRIM25 multimerization inhibition, the RIG-I-TRIM25 interaction disruption, and consequent the IRF3 and TBK1 phosphorylation impediment. Overexpression of SADS-CoV N protein facilitated the replication of VSV-GFP by suppressing IFN-β production. Our results demonstrate that SADS-CoV N suppresses the host IFN response, thus highlighting the significant involvement of TRIM25 in regulating antiviral immune defenses.

**Supplementary Information:**

The online version contains supplementary material available at 10.1186/s13567-024-01303-z.

## Introduction

Coronaviruses (CoVs) infect various animal species and pose a significant threat to both public health and economies [1]. Over the last two decades, severe acute respiratory syndrome coronavirus-1 (SARS-CoV-1), Middle East respiratory syndrome coronavirus (MERS-CoV) and SARS-CoV-2, have caused severe respiratory disease in humans [2]. As an important livestock species, pigs have been particularly susceptible to severe CoV diseases, thus resulting in substantial economic effects [3]. Porcine epidemic diarrhea virus (PEDV), porcine hemagglutinating encephalomyelitis virus (PHEV), porcine deltacoronavirus (PDCoV), transmissible gastroenteritis virus (TGEV), and porcine respiratory coronavirus (PRCV) are five distinct swine CoVs.

The sixth identified porcine coronavirus, swine acute diarrhea syndrome coronavirus (SADS-CoV), was responsible for two significant outbreaks in China [4]. SADS-CoV, classified as a swine enteric alpha-CoV, induces swine acute diarrhea syndrome in piglets and leads to vomiting, severe diarrhea and weight loss. The mortality rates of 5-day-old piglets affected by SADS-CoV can reach 90% [5]. SADS-CoV (family *Coronaviridae*, genus *alphacoronavirus*) is an enveloped, single-stranded positive-sense RNA virus. The genome of SADS-CoV has a typical CoV structure, seven open reading frames encoding four structural proteins, 16 non-structural proteins and an accessory protein [6]. Nucleocapsid (N) proteins have important functions in the transcription, replication, and assembly phases of viruses [7]. N proteins can also act as IFN antagonists. For example, the N protein of PDCoV, PEDV, SARS-CoV-1, SARS-CoV-2 and Mouse hepatitis virus (MHV) suppress IFN production through different mechanisms [8–11].

The innate immune response, as the initial barrier against pathogens, encompasses the type I interferon (IFN-I) signaling pathway, which plays an essential role in defending against viral infections [12]. After the identification of viral RNA within the cytoplasm, retinoic acid-inducible gene I (RIG-I)-like receptors (RLR) initiate the antiviral response through the activation of signaling cascades [13, 14]. To date, three members of the RLR family have been described: RIG-I, melanoma differentiation-associated gene 5 (MDA5), and laboratory of genetics and physiology 2 (LGP2) [15]. RIG-I is critical for the recognition of most single-stranded RNA (ssRNA) viruses, including all negative-strand RNA viruses and some positive-strand RNA viruses, such as coronavirus [16, 17]. RIG-I comprises N-terminal two caspase activation and recruitment domains (2CARDs), a central DECH box ATPase domain, and a C-terminal regulatory/repressor domain (RD). The ATPase and RD domains of RIG-I recognize viral RNAs, exposing the 2CARDs, which interact with tripartite motif-containing protein 25 (TRIM25). TRIM25, a 71-kDa E3 ubiquitin ligase, has four domains: an N-terminal Really Interesting New Gene (RING) domain, two B-boxes, a coiled-coil dimerization domain (CCD), and a C-terminal SPRY domain. TRIM25 is involved in various cellular processes, such as cell proliferation, differentiation and death, and implicated in innate immune signaling pathways [18]. In the absence of an RNA ligand, RIG-I is held in a closed conformation where the 2CARDs are in association with the RD [19]. However, upon recognition of viral RNA, RIG-I exposes its N-terminal 2CARDs, and TRIM25 triggers the synthesis of lysine 63 (K63)-linked polyubiquitin which binds to the 2CARDs. Subsequently, the oligomerization RIG-I binds with MAVS via CARD-CARD interaction to facilitate IFN-I production [20].

Viruses evade host immune attack by antagonizing antiviral defenses. Specifically, virus-encoded protein impede the innate antiviral responses of the host by selectively targeting the expression of IFN genes or the effector molecules induced by IFN [21]. The N protein, which is encoded by CoVs, is the main IFN antagonist. For example, MERS-CoV N interacts with TRIM25 to suppress both IFN-I and IFN-III production [22]. Similarly, SARS-CoV-1 N protein binds to protein activator of protein kinase R (PACT) and TRIM25 to restrain RIG-I activation [8]. SARS-CoV-2 N protein plays a role in TRIM25 and RIG-I complex by suppressing TRIM25 E3 ligase activity toward RIG-I [9]. PRRSV N protein competitively interferes with TRIM25-RIG-I interaction to inhibit RIG-I ubiquitination. However, the detailed roles of SADS-CoV N protein in suppressing IFN-β production and antiviral gene expression have not been directly investigated.

Here, our findings suggested that TRIM25 is upregulated after SADS-CoV infection and significantly inhibits SADS-CoV infection. We identified that SADS-CoV N protein interacts with TRIM25 CCD domain and RIG-I 2CARDs, which inhibits TRIM25 multimerization and TRIM25-RIG-I interaction, thereby suppresses RIG-I signaling and IFN-β production. The study offered valuable mechanism that may facilitate therapeutic drugs development against *Coronaviridae* family members infection.

## Materials and methods

### Cell culture and virus

Porcine intestinal epithelial cells (IPI-2I) and human embryonic kidney cells (HEK293T) were cultured in Dulbecco’s modified Eagle’s medium (DMEM; Sigma-Aldrich, St Louis, USA) containing 10% fetal bovine serum (Invitrogen, USA) and 1% antibiotic–antimycotic (Invitrogen, USA). The SADS-CoV (GenBank accession No. MF094681) was described previously [23]. Sendai virus (SeV) was purchased from the China Center for Type Culture Collection (Wuhan University, China). The recombinant GFP tagged Vesicular Stomatitis Virus (VSV-GFP) was kindly provided by Prof. Zhigao Bu (Harbin Veterinary Research Institute, China).

### Plasmids and antibodies

Pig full-length TRIM25 (GenBank accession number XM_005656971.3) was cloned from IPI-2I cells cDNA and reconstructed with pCMV-Flag (635,688, Clontech) and pCAGGS-HA (P0166, Miaoling Biology) vectors. The deletion mutants of TRIM25 (RING_1–90 aa, B-boxes_91–200 aa, CCD_180–450 aa, and SPRY_451–630 aa) were constructed on the basis of the full-length TRIM25 plasmid and inserted into the pAcGFP-C1 (632,470, Clontech) vector to generate green fluorescent protein (GFP) fusion proteins. TRIM25 DelCCD (lacking the CCD domain) was cloned into the pCAGGS-HA vector, and we named the recombinant plasmid pHA-TRIM25 DelCCD. GFP-SADS-CoV N and N truncated plasmids were stored in our laboratory as previously described [24]. The plasmids of RIG-I and IFN-β-Luc were stored in our laboratory as previously described [25]. The RIG-I truncated mutants (RIG-IN, RIG-IC and RIG-I 2CARDs) were amplified from the full-length RIG-I and inserted into the pCMV-Flag and pCAGGS-GST vectors to generate the recombinant plasmids Flag-RIG-IN, Flag-RIG-IC and GST-2CARDs, respectively. The sequences of the PCR primers used in this study are listed in Additional file 2. Monoclonal antibodies specific for SADS-CoV N protein were stocked in our laboratory [24]. We purchased antibody against DDDDK (ab205606), HA (ab9110), TBK1 (ab40676), p-TBK1 (ab109272), IRF3 (ab68481), p-IRF3 (ab76493), GFP (ab290), GST (ab138491) or TRIM25 (ab167154) from Abcam (Cambridge, MA, USA). We purchased antibody against Flag (F3165), Myc (M4439) or GAPDH (G9545) from Sigma-Aldrich (St. Louis, MO, USA). RNase A, Alexa Fluor™ 488 or 647 goat anti-mouse IgG (H + L) and Alexa Fluor™ 594 goat anti-rabbit IgG (H + L) secondary antibodies were purchased from Thermo Fisher Scientific (Carlsbad, CA, USA). Poly(I:C) was purchased from InvivoGen (Hong Kong, China).

### RNA interference

Three siRNAs targeting TRIM25 were designed by Shanghai Gene Pharma (Shanghai, China); the target sequences were as follows: siTRIM25-1 (sense, 5'-GGCTCACATTGATGCTTAT-3'), siTRIM25-2 (sense, 5'-GCTGAGGCATAAACTGACT-3'), and siTRIM25-3 (sense,5'-GCGATCACGGCTTTGTCAT-3'). SiTRIM25 and siNC negative control were transfected into IPI-2I cells with Lipofectamine RNAiMAX reagent (13778150, Thermo Fisher Scientific, USA). After 36 h transfection, we infected the cells with SADS-CoV at a multiplicity of infection (MOI) of 0.1. The levels of SADS-CoV N mRNA and protein in the infected cells were detected at 24 hours post-infection (hpi).

### Dual-luciferase reporter assays

We seeded HEK293T cells in 24-well plates, and transfected with luciferase reporter plasmids (IFN-β-Luc) and the indicated plasmid alone or together with SADS-CoV N plasmid for 24 h (pRL-TK *Renilla* luciferase reporter plasmid as an internal control). After 24 h transfection, we test the luciferase activity using a dual luciferase reporter assay kit (E1901, Promega, USA).

### Confocal fluorescence microscopy

The plasmid-transfected HEK293T cells and SADS-CoV -non-infected or -infected Vero E6 cells were fixed with 4% paraformaldehyde (16005, Sigma-Aldrich, USA) at 4 °C for 30 min, and permeabilized with 1% Triton X-100 (T8787, Sigma-Aldrich) at room temperature for 15 min. After 5% skim milk blocking at 37 °C for 2 h, cells were incubated with different primary antibodies at 4 °C overnight. After PBS-Tween-20 (PBS containing 0.05% Tween-20; P1379, Sigma-Aldrich) washing, cells were incubated with Alexa Fluor™ 594/488 conjugated secondary antibody at room temperature for 1 h. After PBST washing, cells were counterstained with 4',6-diamidino-2-phenylindole (DAPI) for 15 min, fluorescence images were directly captured under a LSM880-ZEISS confocal laser scanning microscope equipped with Fast Airyscan (Carl Zeiss AG, Oberkochen, Germany).

### RNA extraction and real-time PCR

Total RNA was extracted with a RNeasy Mini Kit (52906, Qiagen, Germany), and reverse transcription was performed with PrimeScript™ IV 1st strand cDNA Synthesis Mix (6215A, Takara, Japan). Real-time PCR was conducted with TB Green Premix Ex Taq™ II (RR820A, Takara, Japan) on a QuantStudio 5 real-time PCR system (Applied Biosystems, Carlsbad, USA). The fold change in gene expression levels was calculated with the comparative CT (ΔΔC_T_) method as described previously [26]. All experiments were performed in at least triplicate. The primers used in the qRT-PCR assays are listed in Additional file 3.

### Western blotting analysis

Whole cell lysates of IPI-2I cells and HEK293T cells were prepared after SADS-CoV infection or transfection with the indicated plasmids in six-well plates. The contents of each well were lysed with RIPA lysis buffer (R0278, Sigma-Aldrich, USA) with 1 mM PMSF (ST506-2, Beyotime) on ice for 30 min. After centrifuging at 12 000 × *g* at 4 °C for 20 min, sodium dodecyl sulfate polyacrylamide gel electrophoresis (SDS-PAGE) loading buffer was added to the supernatant and boiled for 10 min. Proteins were separated with 12.5% SDS-PAGE, then transferred to nitrocellulose membranes at 300 mA for 120 min. After 5% skim milk blocking for 2 h, the membranes were incubated with different primary antibodies at 4 °C for 6–8 h, and then incubated with IRDye 800CW goat anti-mouse lgG (H + L) (1:10 000) (926-32210, LiCor BioSciences) or IRDye 680RD goat anti-rabbit lgG (H + L) (1:10 000) (926-68071, LiCor BioSciences) for 1 h. The membranes were then visualized with Odyssey infrared imaging system (LiCor BioSciences, USA).

### Native PAGE

HEK293T cells were transfected with HA-TRIM25 in the presence or in the absence of Myc-SADS-CoV N. Twenty-four hours after transfection, the cells were lysed with lysis buffer (87788, Thermo Fisher Scientific) on ice for 30 min and then centrifuged at 12 000 × *g* at 4 °C for 10 min to remove the insoluble fraction. The whole-cell extracts were mixed with native PAGE sample loading buffer (Beyotime) and separated with 8% Native polyacrylamide Gel in running buffer containing 1% sodium deoxycholate at 4 °C, as previously described [27]. After electrophoresis, proteins were transferred to nitrocellulose membranes and analyzed by Western blotting with anti-HA antibody.

### Immunoprecipitation

We performed the immunoprecipitation assays as described previously [28]. Plasmid-transfected HEK293T cells were lysed with IP lysis buffer (87788, Thermo Fisher Scientific), which contained 1 mM PMSF and 1 mg/mL protease inhibitor cocktail (04693132001, Roche) on ice for 30 min. Five percent of the cell lysate was collected as input, and the remainder was incubated with the indicated primary antibody at 4 °C overnight, then precipitated with Protein A/G agarose beads (78609, Thermo Fisher Scientific) for 6 h. After five times washing, the beads were collected and analyzed with Western blotting.

### Flow cytometry analysis

After 24 h transfection with Myc-SADS-CoV N, HEK293T cells were infected with VSV-GFP for 12 h. Subsequently, we harvested and resuspended the cells in PBS. Basing on the background signal emitted by uninfected cells, cells were subjected to gating for GFP signals. Fluorescence intensity was tested through BD FACSCalibur instrument. The data analysis was performed in FlowJo software.

### Viral titration

IPI-2I cells were infected with SADS-CoV after HA-TRIM25 or siTRIM25 transfection. The culture supernatants were collected at 24 hpi. Vero E6 cells were infected with tenfold serial dilutions of each supernatant. At 4–6 days post-infection, cytopathic effects in cells were observed through microscopy. We used the Spearman-Kärber method to calculate the median tissue culture infective dose (TCID_50_).

### Experimental infection of piglets

We performed the piglets infection experiment as described previously [23]. We randomly separated six 3 days old specific pathogen free (SPF) piglets into the challenge and control group. The challenge group was orally infected with 5 × 10^4^ TCID_50_ of SADS-CoV, whereas the control group was orally infected with the same volume DMEM. We recorded the clinical symptoms (vomiting and diarrhea), and euthanized all piglets at 36 hpi. Intestinal tissues were collected for Western blotting and qRT-PCR analyses.

### Statistical analysis

The figures display the mean and standard deviation (SD) of results obtained from three independent experiments. Data were analyzed in Graph Pad Prism 8.0. Error bars represent the mean ± SD. *P* values were calculated using two-tailed unpaired Student’s *t*-test.

## Results

### Upregulation of TRIM25 by SADS-CoV infection in vitro and in vivo

**Figure 1:**
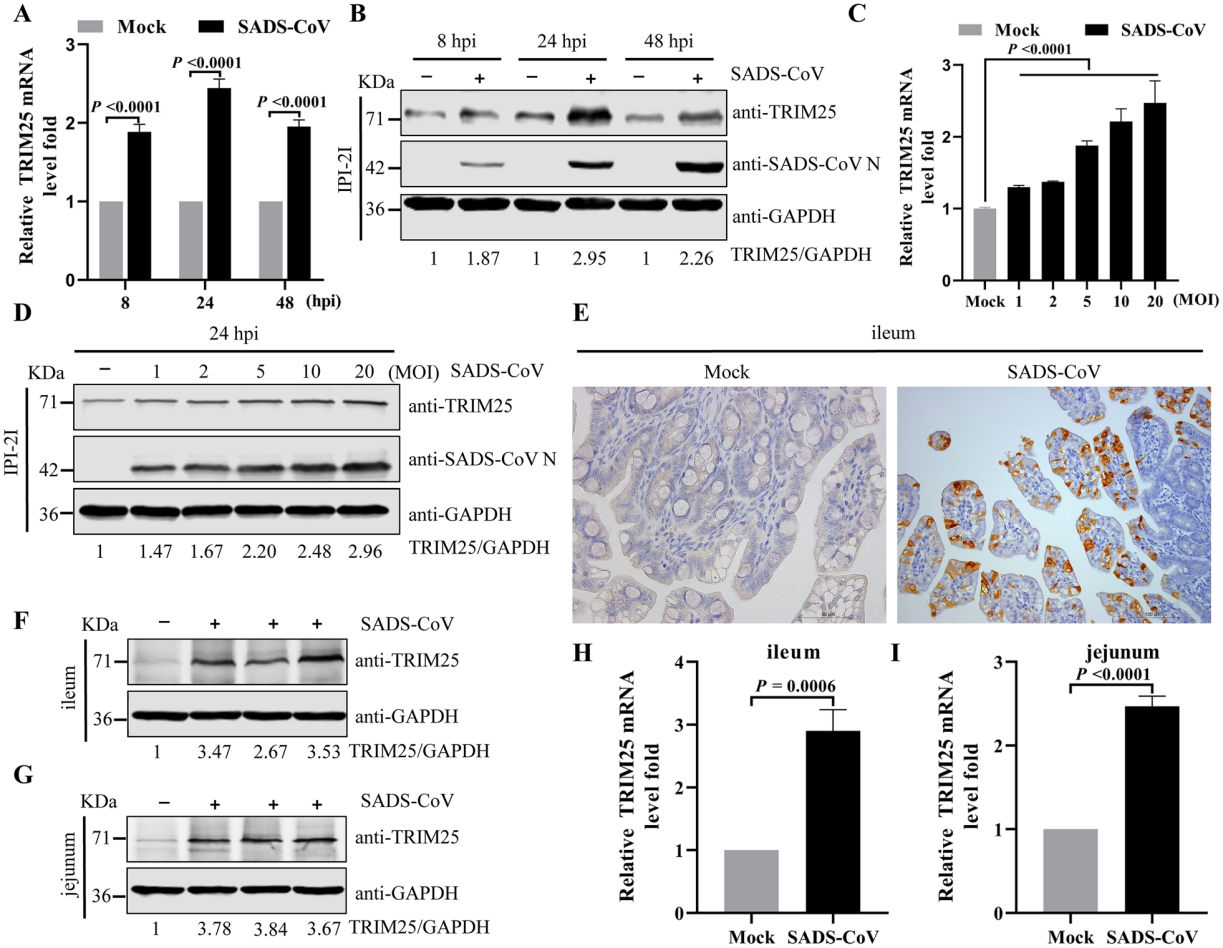
**SADS-CoV infection upregulates TRIM25 expression**. **A** and **C** Relative expression levels of TRIM25 were quantified by qRT-PCR. IPI-2I cells were either mock infected or infected with SADS-CoV 0.1 MOI at different times (A) or different MOIs at 24 hpi (C). Total RNA was extracted, and TRIM25 mRNA levels were evaluated with qRT-PCR. **B** and **D** Relative expression levels of TRIM25 were quantified by Western blotting. IPI-2I cells were either mock infected or infected with SADS-CoV 0.1 MOI at different times (B) or different MOIs at 24 hpi (D). The cell lysates were collected, and the expression of TRIM25 was tested by Western blotting. The densities of TRIM25 bands relative to GAPDH were calculated. The value of each negative control cells group was normalized to one. **E** Representative microphotographs of viral antigen immunochemical staining in SADS-CoV -non-infected and -infected ileum tissues (Bar: 50 µm). **F-I** SADS-CoV infection upregulates TRIM25 expression in vivo. **F** and **G** Western blotting analysis of the protein levels of TRIM25 in ileum (F) and jejunum (G) samples from SADS-CoV -non-infected and -infected piglets at 36 hpi. The densities of TRIM25 bands relative to GAPDH were calculated. The value of control piglet was normalized to one. **H** and **I** qRT-PCR analysis of the mRNA expression levels of TRIM25 in ileum (H) and jejunum (I) samples from SADS-CoV -non-infected and -infected piglets at 36 hpi. *P* values were calculated using two-tailed unpaired Student’s *t*-test.

Previous studies have indicated that multiple virus infection affect the expression of TRIM25 [29, 30]. To determine the expression level and potential role of TRIM25 in SADS-CoV infection, we infected IPI-2I cells with 0.1 MOI SADS-CoV. After qRT-PCR and Western blotting analysis, we observed marked upregulation of both TRIM25 mRNA and protein at 8, 24, and 48 hpi (Figures 1A and B). In addition, SADS-CoV significantly upregulated the expression of TRIM25 with increasing SADS-CoV MOI values (Figures 1C and D). To explore whether SADS-CoV infection might upregulate the expression of TRIM25 in vivo, the ileum and jejunum tissue from SADS-CoV-infected piglets at 36 hpi were dissected. SADS-CoV infection was confirmed by immunohistochemistry with a specific mAb (3E9) against the SADS-CoV N protein (Figure 1E). The protein levels of TRIM25 in SADS-CoV-infected ileum and jejunum tissue exhibited a significant increase compared with the control group (Figures 1F and G). Meanwhile, TRIM25 mRNA levels were higher in infected group (Figures 1H and I). Together, these results suggested that TRIM25 was upregulated by SADS-CoV infection in vitro and in vivo.

### TRIM25 affects SADS-CoV replication

**Figure 2:**
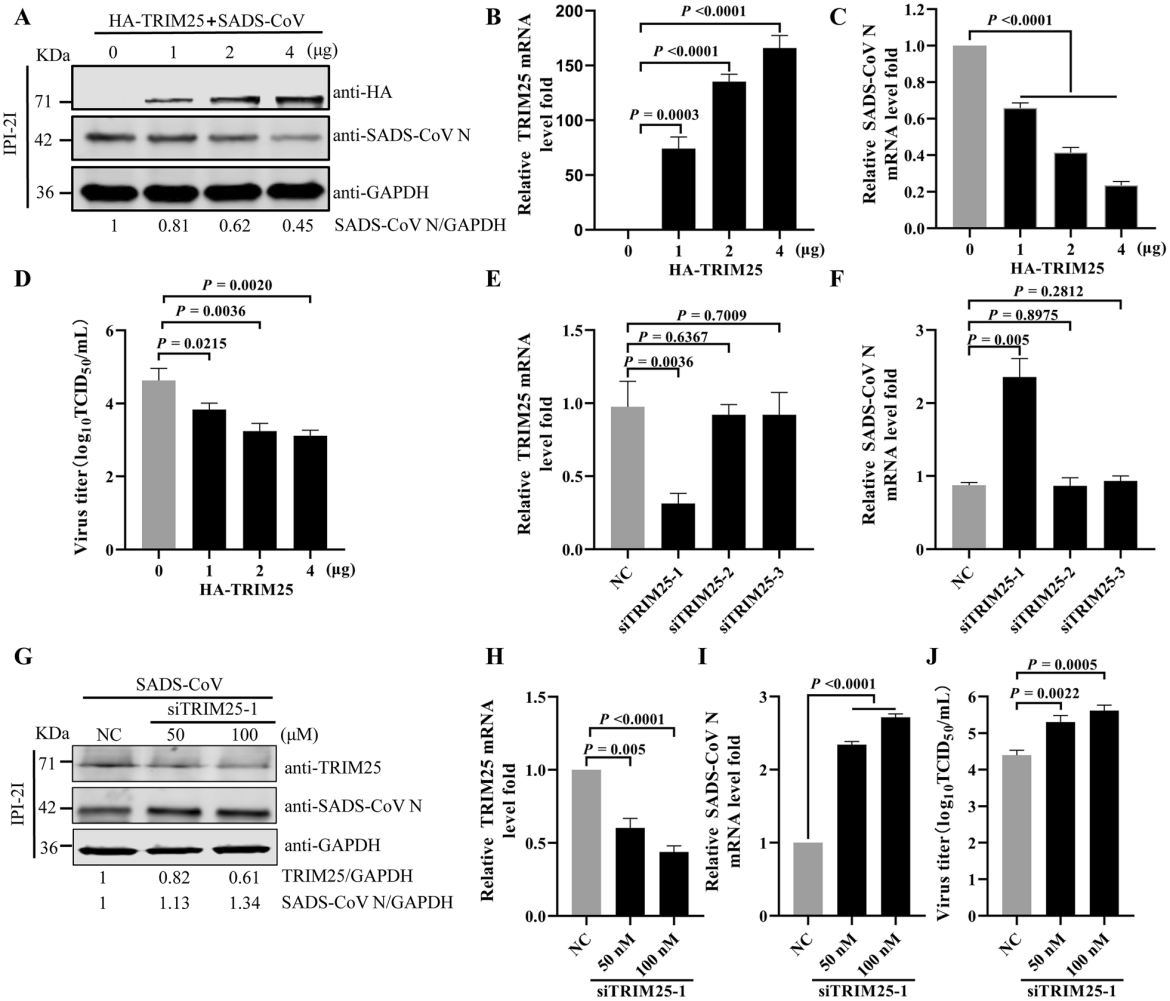
**TRIM25 affects the replication of SADS-CoV. A**–**D** Overexpression of TRIM25 inhibits SADS-CoV replication. IPI-2I cells were transfected with HA-tagged TRIM25 (0, 1, 2, or 4 μg) for 24 h and then infected with SADS-CoV at an MOI of 0.1 for 24 h.** A** The protein expression of TRIM25 and SADS-CoV N was detected by Western blotting. The densities of SADS-CoV N bands relative to GAPDH were calculated. The value of HA-TRIM25 (0 μg) group was normalized to one. **B** Overexpression of TRIM25 was verified by qRT-PCR. **C** The mRNA levels of SADS-CoV N were determined by qRT-PCR. **D** The SADS-CoV TCID_50_ in the supernatants was titrated on Vero E6 cells. **E-J** Knockdown of TRIM25 enhanced SADS-CoV replication. **E** and **F** IPI-2I cells were transfected with siTRIM25-1, siTRIM25-2, siTRIM25-3, or siNC (negative control) at 50 nM for 36 h and subsequently infected with SADS-CoV at an MOI of 0.1 for 24 h. The mRNA levels of TRIM25 (E) and SADS-CoV N (F) in TRIM25 knockdown cells were determined by qRT-PCR. **G-J** IPI-2I cells were transfected with siNC or siTRIM25-1 (50 nM or 100 nM) for 36 h and subsequently infected with SADS-CoV at an MOI of 0.1 for 24 h. **G** The protein expression of TRIM25 and SADS-CoV N was detected by Western blotting. The densities of TRIM25 and SADS-CoV N bands relative to GAPDH were calculated. The value of negative control cells group was normalized to one. **H** The mRNA levels of TRIM25 were verified by qRT-PCR. **I** The mRNA levels of SADS-CoV N were determined by qRT-PCR. **J** The SADS-CoV TCID_50_ in the supernatants was titrated on Vero E6 cells. *P* values were calculated using two-tailed unpaired Student’s *t*-test.

To determine the role of TRIM25 in SADS-CoV infection, IPI-2I cells were transfected with TRIM25 expression plasmid and infected with SADS-CoV. Western blotting showed that the N protein levels of SADS-CoV significantly decreased in a dose-dependent manner by TRIM25 overexpression (Figure 2A). In agreement with the observed changes in protein levels, increased TRIM25 expression was associated with decreases in mRNA levels of N protein ranging from 34.2% to 76.43% in IPI-2I cells, according to qRT-PCR (Figures 2B and C). Furthermore, TCID_50_ assays indicated that the titer of released virus decreased after TRIM25 overexpression (Figure 2D). Together, these results indicated that TRIM25 upregulation inhibited SADS-CoV replication.

To further ascertain the effect of TRIM25 on SADS-CoV replication, three siRNAs targeting TRIM25 were synthesized. The qRT-PCR results showed that TRIM25 mRNA levels decreased significantly after transfection of IPI-2I cells with siTRIM25-1 (Figure 2E). In contrast, the mRNA level of the viral N gene exhibited an increase (Figure 2F). IPI-2I cells was transfected with siTRIM25-1 at different concentrations, and subsequently infected with 0.1 MOI SADS-CoV for 24 h. As shown in Figure 2G, siTRIM25-1 promoted SADS-CoV propagation. The qRT-PCR results indicated lower TRIM25 mRNA levels (Figure 2H) and higher SADS-CoV N mRNA levels (Figure 2I) than observed in the siNC group. Furthermore, TCID_50_ assays indicated that the viral titer was greater after TRIM25 knockdown than control siNC transfection (Figure 2J). These results indicated that TRIM25 silencing promoted SADS-CoV replication. Together, our results suggested that TRIM25 acts as an antiviral factor inhibiting SADS-CoV infection.

### TRIM25 interacts with SADS-CoV N

**Figure 3:**
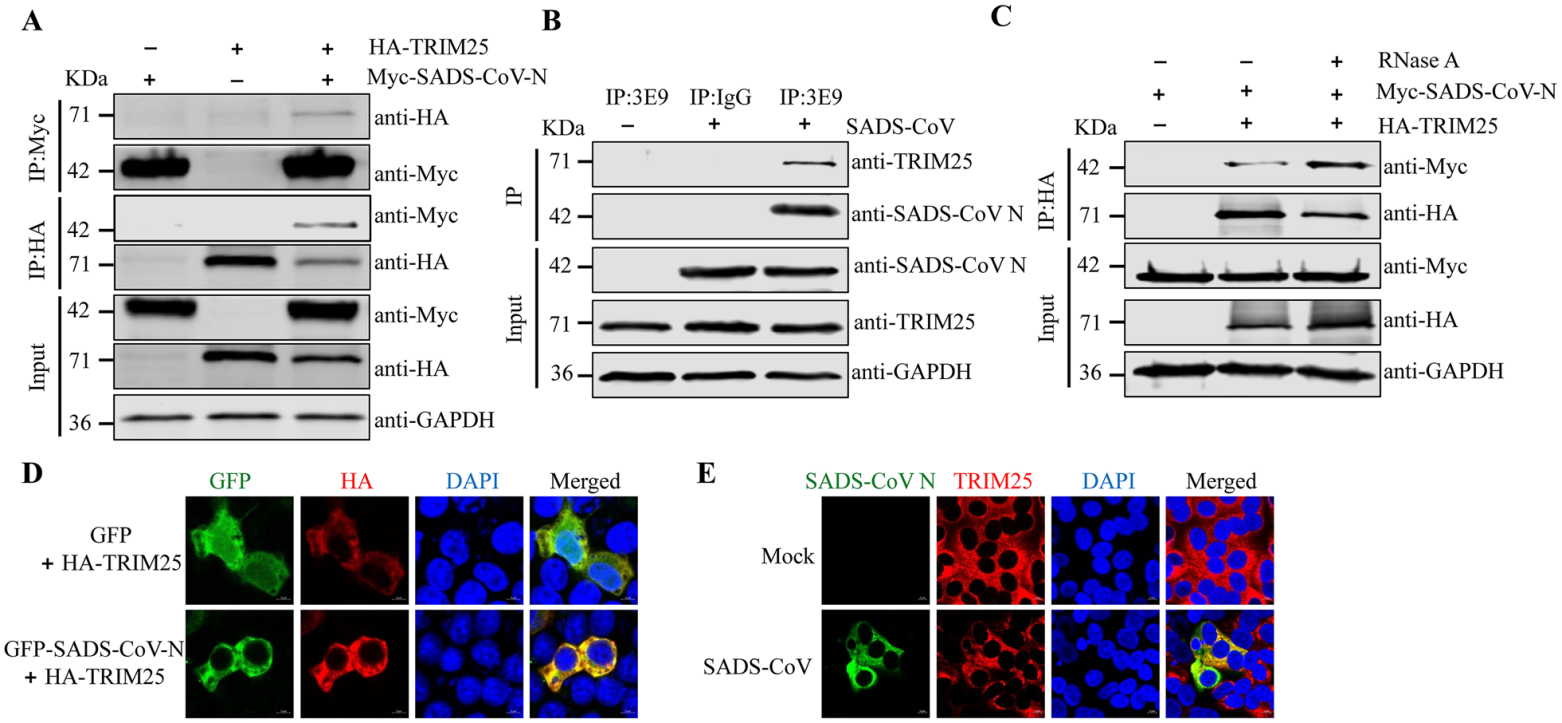
**TRIM25 interacts with SADS-CoV N protein. A** Co-IP analysis of the interaction between SADS-CoV N protein and TRIM25 in HEK293T cells. HEK293T cells were transfected with HA-TRIM25, Myc-SADS-CoV N, or empty vector. At 24 h post-transfection, cell lysates were subjected to immunoprecipitation with anti-Myc or anti-HA antibodies, and subsequent immunoblotting with anti-Myc or anti-HA antibodies. **B** The interaction of endogenous TRIM25 and SADS-CoV N protein was detected with Co-IP assays. IPI-2I cells were infected with SADS-CoV at an MOI of 0.1 for 24 h. Cell lysates were collected and then incubated with anti-mouse IgG or anti-SADS-CoV N mAb (3E9), and detected with the indicated antibodies by Western blotting. **C** SADS-CoV N protein binds TRIM25 in a manner independent of RNA. HA-TRIM25, Myc-SADS-CoV N, or empty vector were co-expressed in HEK293T cells. At 24 h post-transfection, the cell lysates were either immunoprecipitated with antibody against the HA-tag or treated with 100 mg/mL RNase A on ice for 2 h before IP. SADS-CoV N and TRIM25 were detected by Western blotting with anti-Myc or anti-HA antibodies, respectively. **D** and **E** Co-localization of TRIM25 and SADS-CoV N.** D** HEK293T cells were co-transfected with HA-TRIM25, GFP-SADS-CoV N, or GFP empty vector plasmids. The GFP and GFP-SADS-CoV N were in green, and the TRIM25 fusion protein was in red. Merged images were also presented, and the positions of the nuclei were indicated by DAPI (blue) staining in the merged images. **E** Vero E6 cells were mock infected or infected with SADS-CoV at an MOI of 0.1 for 24 h. Cells were fixed and co-stained with antibodies directed against SADS-CoV N (green) and TRIM25 (red). The cells were then counterstained with DAPI (blue) and observed with a confocal laser scanning microscope.

CoVs N protein is highly abundant in infected cells and has a crucial function in viral transcription and assembly [31, 32]. SARS-CoV-1 and PRRSV N protein inhibit IFN-I production by competitively interfering with TRIM25-RIG-I interaction and suppressing RIG-I ubiquitination [33, 34]. SADS-CoV, SARS-CoV-1, and PRRSV belong to the *Nidovirales* order, and TRIM25 also has important roles in the cellular anti-SADS-CoV response, we next investigated whether SADS-CoV N protein might interact with TRIM25 and antagonize its antiviral effects. To test this possibility, we assessed the interaction between TRIM25 and SADS-CoV N protein. We found that Myc-tagged SADS-CoV N interacted with HA-tagged TRIM25 protein (Figure 3A). In addition, we immunoprecipitated virus-infected IPI-2I cells with anti-SADS-CoV N mAb (3E9), then performed Western blotting. As shown in Figure 3B, endogenous TRIM25 co-immunoprecipitated with SADS-CoV N protein. To eliminate the effects of RNA interference on the interaction between TRIM25 and the N protein of SADS-CoV, we used RNase A to remove any RNA molecules present in the experimental system. The SADS-CoV N-TRIM25 interaction was not impeded in the presence of RNase A, suggesting that the interaction between TRIM25 and SADS-CoV N protein did not rely on RNA (Figure 3C). Additionally, the co-localization of SADS-CoV N and TRIM25 was observed through indirect immunofluorescence (Figures 3D and E). These data indicated that TRIM25 interacted with the SADS-CoV N protein.

### Amino acids 215–249 of SADS-CoV N protein interact with TRIM25 CCD domain and inhibit CCD-dependent TRIM25 oligomerization

**Figure 4:**
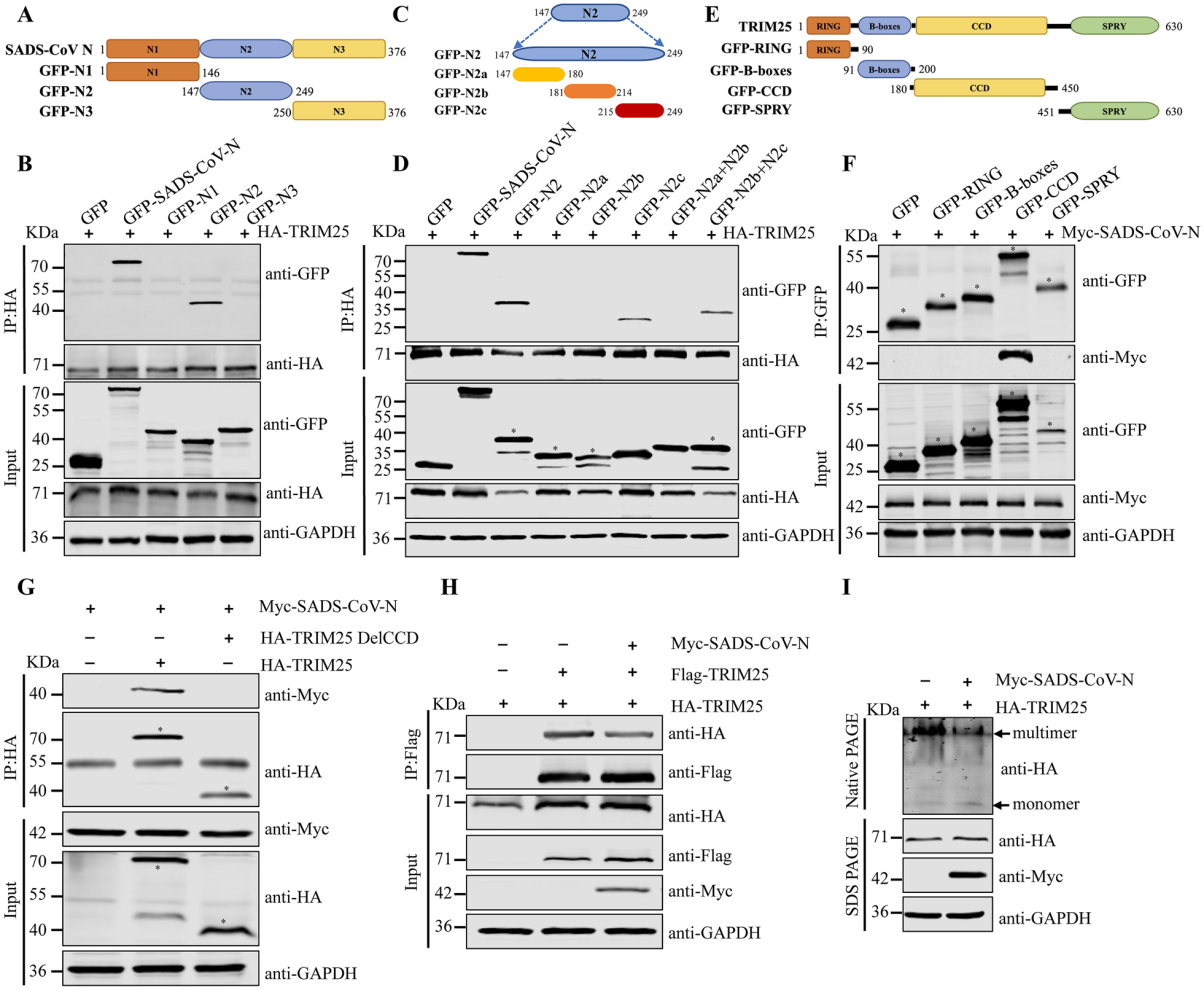
**SADS-CoV N protein inhibits CCD-dependent TRIM25 oligomerization. A** and **C** Schematics of SADS-CoV N and the SADS-CoV N2 domain truncations. **B** and **D** The domain of SADS-CoV N responsible for interaction with TRIM25 was identified. HA-TRIM25 together with GFP empty vector, GFP-SADS-CoV N, or SADS-CoV N truncations were co-expressed in HEK293T cells for 24 h. Whole cell lysates were collected and subjected to IP with anti-HA antibody, followed by immunoblotting with anti-GFP or anti-HA antibodies. **E** Schematics of TRIM25 truncations. **F** The domain of TRIM25 responsible for interaction with SADS-CoV N was identified. Myc-SADS-CoV N, GFP empty vector, or TRIM25 truncations with GFP were co-expressed in HEK293T cells. At 24 h post-transfection, the cell lysates were immunoprecipitated with antibody against GFP. SADS-CoV N and TRIM25 truncations were detected by Western blotting with anti-Myc or anti-GFP antibodies, respectively. **G** SADS-CoV N did not interact with mutants of TRIM25 lacking the CCD domain. HEK293T cells were co-transfected with Myc-SADS-CoV N together with empty vector, HA-TRIM25, or HA-TRIM25 DelCCD. At 24 h post-transfection, whole cell lysates were collected and used for IP with anti-HA antibody, followed by immunoblotting with anti-Myc or anti-HA antibodies. **H** SADS-CoV N protein inhibited the interaction of differently labeled TRIM25. HEK293T cells were transfected with Myc-SADS-CoV N together with empty vector, Flag-TRIM25, or HA-TRIM25. At 24 h post-transfection, whole cell lysates were collected and used for IP with anti-Flag antibody, followed by immunoblotting with anti-Myc, anti-HA, and anti-Flag antibodies. **I** SADS-CoV N protein expression interferes with TRIM25 multimerization. HEK293T cells were transfected with HA-TRIM25 in the presence or in the absence of Myc-SADS-CoV N for 24 h. The cell lysates were separated with Native PAGE followed by Western blotting to detect the expression of individual proteins.

To clarify the specific region of SADS-CoV N protein required for SADS-CoV N-TRIM25 interaction, we co-transfected HEK293T cells with GFP-tagged truncated fragments of SADS-CoV N protein (aa 1–146, 147–249, and 250–376) with HA-TRIM25. Co-IP assays revealed that the N2 domain of SADS-CoV N (aa 147–249) was the essential region for SADS-CoV N-TRIM25 interaction (Figures 4A and B). In contrast, the other truncated regions of SADS-CoV N protein were not responsible for this association. To further determine the necessary amino acid residues in SADS-CoV N2 domain (aa 147–249) directing the SADS-CoV N-TRIM25 interaction, we constructed a series of overlapping recombinant N2 truncated mutant proteins and determined their interaction with TRIM25 through Co-IP assays (Figure 4C). As shown in Figure 4D, GFP-N2c and GFP-N2b + N2c were responsible for the interaction with TRIM25. These results further suggested that S^215^PAPAPKPARKQMDKPEWKRVPNSEEDVRKCFGPR^249^ of N protein is the key region for SADS-CoV N-TRIM25 interaction. To enhance understanding of the SADS-CoV N-TRIM25 interaction, we comprehensively analyzed the structural domains necessary for TRIM25 binding to SADS-CoV N proteins, by using a series of GFP-tagged deletion mutants (Figure 4E). As depicted in Figure 4F, the CCD domain of TRIM25 interacted with SADS-CoV N, whereas the other domains did not show any interaction. Furthermore, the CCD deletion TRIM25 mutant (TRIM25 Del CCD) was unable to bind SADS-CoV N protein (Figure 4G). The coiled coil region of the TRIM family proteins primarily participated in homo-oligomeric interactions, and promoted the formation of a hypersecondary structure with multiple a helices [35]. Mutants lacking the CCD region of TRIM25 cannot multimerize [36]. Given that SADS-CoV N interacted with the CCD domain of TRIM25, we next tested whether SADS-CoV N might interfere with TRIM25 multimerization. Indeed, SADS-CoV N expression effectively inhibited CCD-mediated TRIM25 multimerization (Figures 4H and I).

### SADS-CoV N protein interferes with TRIM25-RIG-I interaction

**Figure 5:**
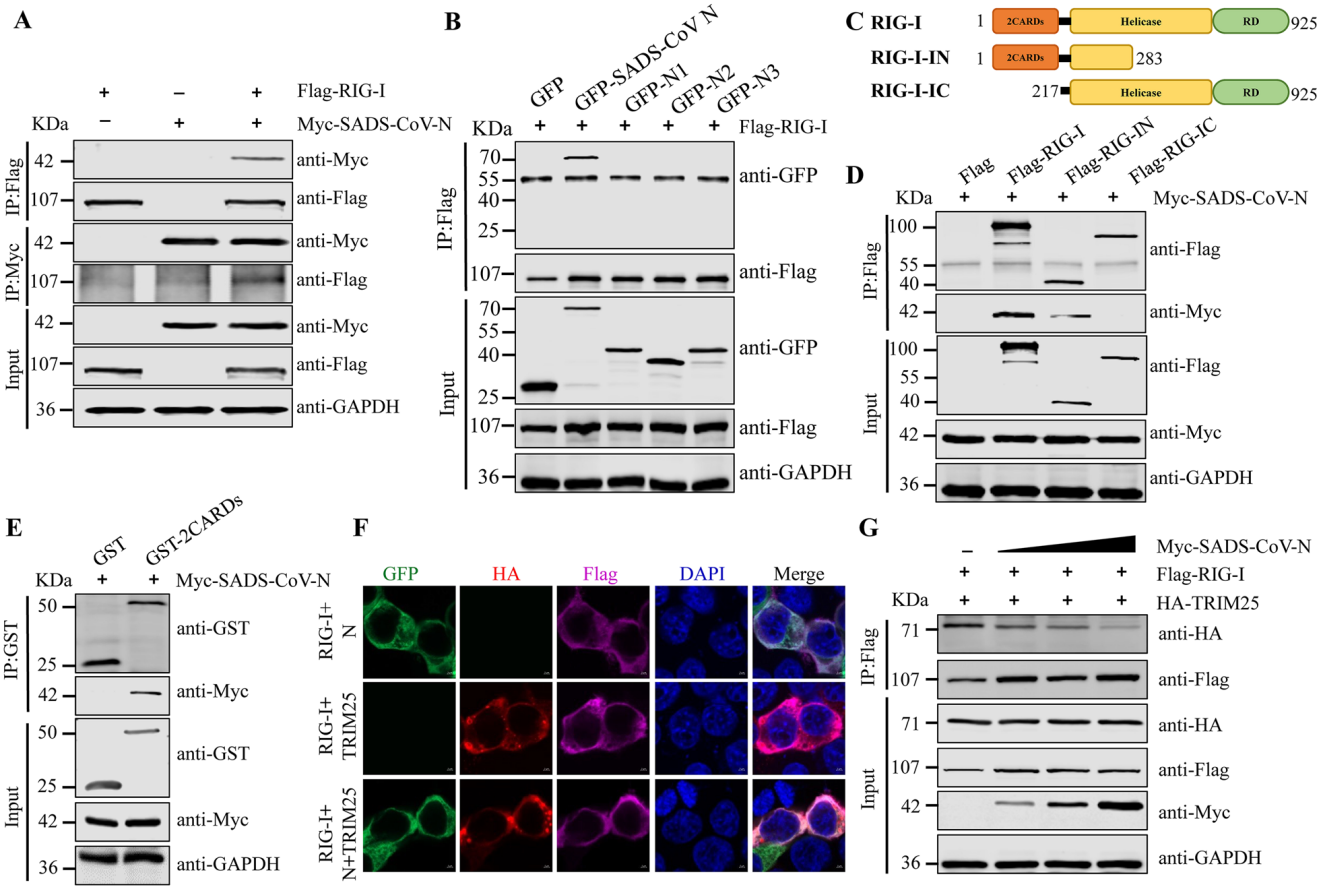
**SADS-CoV N protein interferes with the interaction between TRIM25 and RIG-I. A** RIG-I interacts with SADS-CoV N protein. HEK293T cells were transfected with Flag-RIG-I, Myc-SADS-CoV N, or empty vector. At 24 h post-transfection, cell lysates were subjected to immunoprecipitation with anti-Myc or anti-Flag antibodies, and subsequent immunoblotting with anti-Myc or anti-Flag antibodies. **B** The domain of SADS-CoV responsible for interaction with RIG-I was identified. **C** Schematics of RIG-I truncations. **D** The domain of RIG-I responsible for interaction with SADS-CoV N was identified. **E** RIG-I 2CARDs interacts with SADS-CoV N protein. HEK293T cells were transfected with GST or GST-RIG-I 2CARDs together with Myc-SADS-CoV N. The cell lysates were collected and then immunoprecipitated with anti-GST antibody. The immunoprecipitates were detected by Western blotting with the indicated antibodies. **F** Co-localization of RIG-I with TRIM25 and SADS-CoV N. HEK293T cells were co-transfected with Flag-RIG-I and HA-TRIM25 or GFP-SADS-CoV N for 24 h. The cells were incubated with mouse anti-Flag antibody and rabbit anti-HA antibody before being stained with DAPI. **G** SADS-CoV N protein dose-dependently inhibited RIG-I interaction with TRIM25. HEK293T cells were co-transfected with Flag-RIG-I and HA-TRIM25 or Myc-SADS-CoV N for 24 h. The cell lysates were collected and then immunoprecipitated with anti-Flag antibody. The immunoprecipitates were detected by Western blotting with the indicated antibodies.

Given that TRIM25 is a RIG-I regulatory partner, our subsequent studies were designed to determine SADS-CoV N-RIG-I interaction. To test this possibility, we investigated the SADS-CoV N-RIG-I interaction through Co-IP. On the basis of precipitation with antibodies against Flag or Myc tag, we determined that RIG-I interacted with SADS-CoV N protein (Figure 5A). Further Co-IP assays indicated that only full-length SARS-CoV N interacted with RIG-I (Figure 5B). We also generated Flag-tagged N-terminal (Flag-RIG-IN) and C-terminal (Flag-RIG-IC) constructs and assessed them in Co-IP experiments (Figure 5C). Co-IP assays revealed that the SADS-CoV N protein interacted with Flag-RIG-I and Flag-RIG-IN, but not Flag-RIG-IC (Figure 5D). To further establish that the RIG-I 2CARDs was sufficient for interaction with SADS-CoV N, GST-RIG-I-2CARDs and Myc-SADS-CoV N were co-transfected in HEK293T cells. The results indicated that the N-terminal 2CARDs of RIG-I was crucial for SADS-CoV N-RIG-I interaction (Figure 5E). To explore the localization of the N/RIG-I, and TRIM25/RIG-I, and N/TIRM25/RIG-I complexes, we performed confocal microscopy. Imaging showed that RIG-I was colocalized with SADS-CoV N and TRIM25 in the cytoplasm (Figure 5F). These results further suggested that SADS-CoV N interacts with the RIG-I.

Study showed that TRIM25 SPRY domain interacted with RIG-I, which induced K63-linked ubiquitination of the RIG-I N-terminal 2CARDs, thereby markedly activating RIG-I downstream signaling [37]. Given the association of the RIG-I N-terminal 2CARDs with SADS-CoV N protein, we speculated that the binding of RIG-I to TRIM25 might be affected by SADS-CoV N protein. To examine this possibility, we co-expressed HA-TRIM25 and Flag-RIG-I in HEK293T cells, both with and without Myc-SADS-CoV N. Co-IP assays revealed that overexpression of SADS-CoV N protein attenuated TRIM25-RIG-I interaction, which was a dose-dependent manner (Figure 5G). Thus, SADS-CoV N protein competitively binds RIG-I N-terminal 2CARDs and interferes with the TRIM25-RIG-I interaction.

### SADS-CoV N protein suppresses IFN-β production

**Figure 6:**
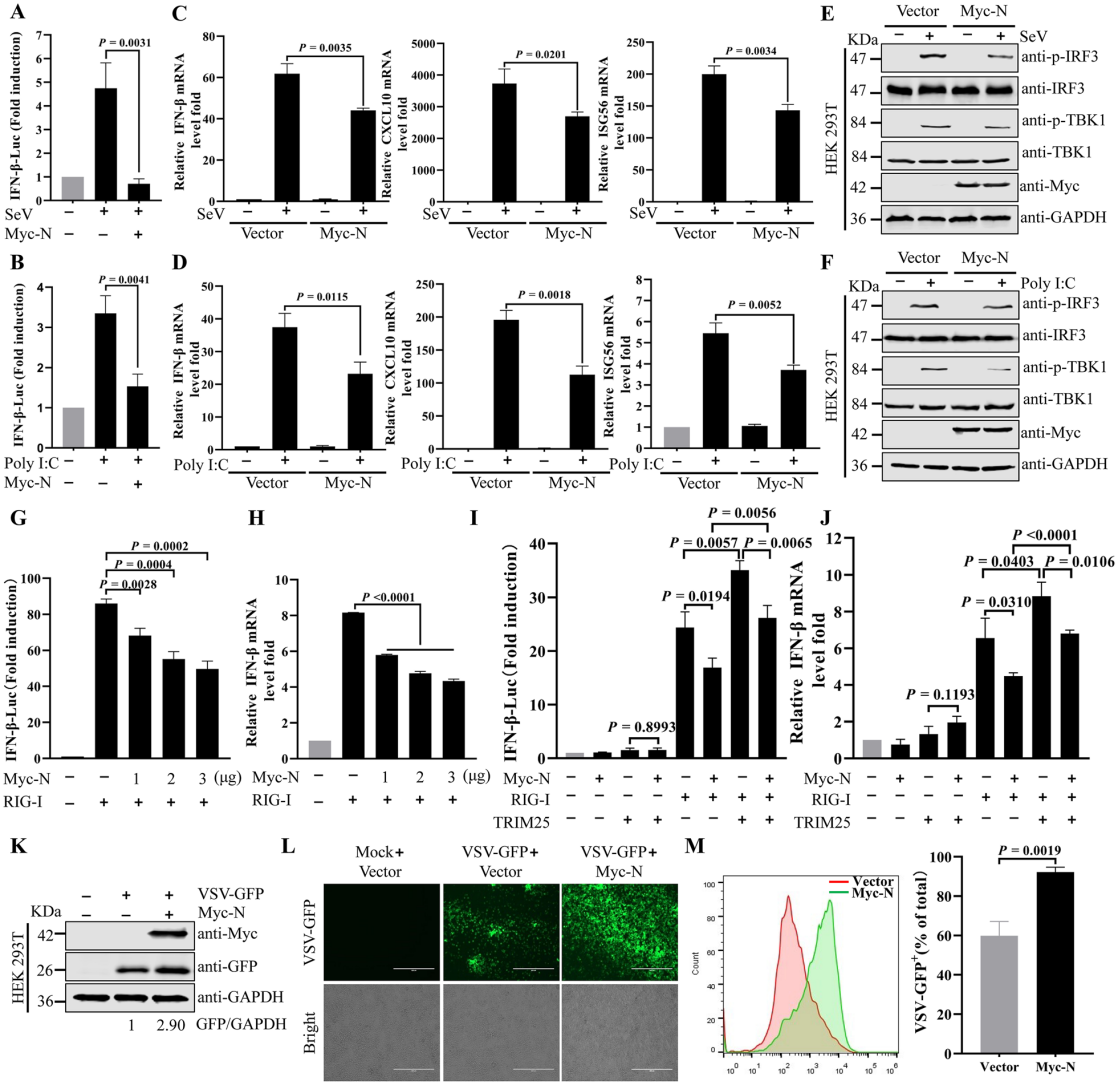
**SADS-CoV N protein suppresses IFN-β production in HEK293T. A** and **B** SADS-CoV N inhibited the SeV and poly(I:C)-mediated IFN-β promoter activity in HEK293T cells. HEK293T cells were transfected with plasmid for SADS-CoV N expression (Myc-N) and IFN-β-Luc plasmid for 24 h. Cells were infected with SeV (A) or stimulated with poly(I:C) (B) for 12 h. Luciferase assays were performed with a dual luciferase reporter assay kit. **C** and **D** SADS-CoV N inhibits the transcription of IFN-β, CXCL10, and ISG56 after SeV infection and poly(I:C) transfection in HEK293T cells. HEK293T cells were transfected with empty vector or Myc-N for 24 h. Cells were stimulated with SeV infection C or poly(I:C) transfection (D) for 12 h. The relative mRNA levels of IFN-β, CXCL10, and ISG56 were evaluated by qRT-PCR. **E** and **F** SADS-CoV N inhibits phosphorylation of IRF3 and TBK1 stimulated by SeV and poly(I:C) in HEK293T cells. HEK293T cells were transfected with Myc-N or empty vector for 24 h. Cells were stimulated with SeV infection (E) or poly(I:C) transfection (F) for 12 h. The cell lysates were subjected to Western blotting analysis. **G** SADS-CoV N suppresses the RIG-I-induced activation of luciferase reporters of IFN-β. HEK293T cells were co-transfected with IFN-β-Luc, Flag-RIG-I, pRL-TK plasmid, and Myc-N for 24 h. Luciferase activity was assessed. **H** SADS-CoV N inhibits mRNA levels of IFN-β induced by RIG-I. HEK293T cells were co-transfected with Flag-RIG-I and Myc-N for 24 h. Cells were harvested for qRT-PCR analysis. **I** and **J** SADS-CoV N inhibits TRIM25-mediated RIG-I signaling enhancement. **I** HEK293T cells were co-transfected with IFN-β-Luc, and Myc-N, HA-TRIM25 or Flag-RIG-I expression plasmid for 24 h. Luciferase assays were performed with a dual luciferase reporter assay kit. **J** HEK293T cells were transfected with Myc-N, Flag-RIG-I, or HA-TRIM25 expression plasmid for 24 h. The IFN-β mRNA levels were evaluated with qRT-PCR. **K**–**M** SADS-CoV N facilitates the replication of VSV-GFP. HEK293T cells were transfected with Myc-N and then infected with VSV-GFP for 12 h. Fluorescence was analyzed through Western blotting (K), fluorescence microscopy (L), and flow cytometry (M). *P* values were calculated using two-tailed unpaired Student’s *t*-test.

To explore whether the presence of SADS-CoV N protein in the RIG-I/TRIM25 complex might inhibit RIG-I downstream signaling and IFN-β production, HEK293T were transfected with Myc-N or Myc expression plasmid for 24 h, and then infected with Sendai virus (SeV) or stimulated with poly(I:C) for another 12 h. SADS-CoV N protein inhibited the IFN-β promoter activity inducing by SeV and poly(I:C) (Figures 6A and B). qRT-PCR analysis indicated that the expression of SADS-CoV N protein decreased the SeV- or poly(I:C)-induced transcription of IFN-β, CXCL10, and ISG56 in HEK293T cells (Figures 6C and D) and IPI-2I cells (Additional files 1A and B). Consistently, ectopic expression of SADS-CoV N inhibited the phosphorylation of IRF3 and TBK1 stimulated by SeV and poly(I:C) in HEK293T cells (Figures 6E and F) and IPI-2I cells (Additional files 1C and D). These data indicated that SADS-CoV N protein suppresses IFN-β production. To examine whether SADS-CoV N protein might regulate RIG-I mediated IFN-β activation, HEK293T cells were transfected with firefly luciferase reporter plasmid, empty vector, or SADS-CoV N and RIG-I expression plasmid. The RIG-I induced IFN-β promoter activation was significantly suppressed in a dose-dependent manner by SARS-CoV N protein (Figure 6G). Similarly, N protein of SADS-CoV inhibited RIG-I mediated IFN-β transcription (Figure 6H). Furthermore, luciferase assays and qRT-PCR indicated that TRIM25 further enhanced IFN-β promoter activity and transcription levels by acting on RIG-I; however, SADS-CoV N decreased the TRIM25-mediated RIG-I signaling enhancement (Figures 6I and J). We next investigated whether SADS-CoV N protein might regulate VSV-GFP infection and proliferation. Fluorescence microscopy, flow cytometry, and Western blotting showed that Myc-N plasmid-transfected HEK293T cells, compared with vector-treated cells, facilitated the replication of VSV-GFP, as measured by GFP signaling (Figures 6K–M), thus suggesting that SADS-CoV N inhibits the secretion of antiviral factors. Collectively, these data indicated that SADS-CoV N inhibits the TRIM25-mediated enhancement of RIG-I signaling.

## Discussion

SADS-CoV infection entails a multifaceted interplay between virus and host. Understanding the SADS-CoV and host protein interaction can not only elucidate the mechanism of viral infection but also identify antiviral factors. The E3 ubiquitin ligase tripartite motif-containing protein 25 (TRIM25) plays an important role in antiviral activities. TRIM25 not only directly antagonizes viral replication by degrading viral proteins, but also regulates signal transduction pathways induced by innate immune sensors [29, 38]. During viral infection, TRIM25 mediates lysine 63- linked ubiquitination of the N-terminal 2CARDs of the viral RNA sensor retinoic acid-inducible gene I (RIG-I) to facilitate IFN-I production and antiviral immunity [37]. In the present study, it was determined that the crucial RIG-I activating protein TRIM25 acted as a novel host restriction factor in SADS-CoV infection. Experimental results demonstrated that SADS-CoV N protein interacted with both TRIM25 and RIG-I protein, leading to the inhibition of TRIM25/RIG-I complex formation and the subsequent suppression of IFN-β induction. These findings contribute to a more comprehensive understanding of the intricate regulatory interplay between SADS-CoV and host immune responses.

A multitude of TRIM family proteins have significant functions in the inhibition of viral replication. After viral infection, the mRNA and protein level of several TRIM protein are upregulated; these proteins subsequently act as host factors promoting the production of IFNs and inhibiting viral replication. TRIM14 is upregulated after HSV-1 infection and positively regulates type I IFN signaling [39]. TRIM22, a restriction factor, is upregulated at both the mRNA and protein levels in influenza A virus infected human alveolar epithelial A549 cells [40]. In the current study, SADS-CoV infection upregulated the levels of TRIM25 mRNA and protein in vitro and in vivo (Figure 1). The most prominent role of TRIMs is inhibition of viral replication. TRIM56 impairs hepatitis B virus, PEDV, yellow fever virus, and human coronavirus OC43 infection [41–43]. TRIM5α inhibits human immunodeficiency virus type 1 (HIV-1) infection [44]. We found that upregulation of TRIM25 restricts SADS-CoV infection, whereas downregulation of this protein enhances SADS-CoV infection (Figure 2). Therefore, TRIM25 is a novel host factor that exerts inhibitory effects on SADS-CoV replication.

The N proteins of different coronaviruses are highly conservated and perform multiple functions during viral infection [6]. TRIM25 is targeted by MERS-CoV N [22] and SARS-CoV N protein [33] for immune evasion. Similarly, SADS-CoV N protein also interacts with TRIM25 (Figure 3). The N^S215-R249^ domain of SADS-CoV N was critical for binding TRIM25 in vitro. Despite variations in length and primary sequence among nucleoproteins from different coronaviruses, a conserved three-domain organization has been identified. Notably, domain II of the N protein is highly conserved, as compared with domains I and III [45]. The N^S215-R249^ domain of SADS-CoV N protein is in domain II. TRIM25 consists of a RING-finger domain, two B-box domains, a central CCD, and a C-terminal SPRY domain [38]. SADS-CoV N protein interacts with TRIM25 and targets the TRIM25 CCD, thereby disrupting TRIM25 multimer formation (Figure 4). The formation of TRIM25 multimers acts a pivotal role in RIG-I 2CARDs ubiquitination—a crucial modification necessary for the optimal IFN production in response to viral infection [37]. SADS-CoV N interacts with TRIM25, and inhibits the signal transduction of RIG-I and IFN-β production. The similarity of the observed mechanism to those used by paramyxovirus V [46] and influenza A virus NS1 [36] demonstrates that TRIM25 may be a common viral target for RIG-I antagonism. Interestingly, despite SADS-CoV N and paramyxovirus V both targeting TRIM25, our data indicated that SADS-CoV N utilized a different mechanism to inhibit TRIM25 and RIG-I compared with paramyxovirus V. Paramyxovirus V targets TRIM25 SPRY domain, whereas SADS-CoV N protein interacts with TRIM25 CCD domain. Despite these differences, SADS-CoV N, paramyxovirus V, and influenza A virus NS1 proteins ultimately similarly suppress RIG-I signaling ubiquitination/activation by precluding TRIM25 binding to RIG-I.

Viruses evolving to inhibit antiviral activity induced by IFN is well-documented. For instance, viral protein specifically impedes RIG-I- mediated the signal transduction. Influenza A virus NS1 inhibits the IFN-I induction via the interaction with RIG-I [47]. We found SADS-CoV N interacts with the N-terminal 2CARDs of RIG-I, thus reducing the TRIM25-RIG-I interaction (Figure 5). However, only full-length SARS-CoV N interacts with RIG-I. The structural integrity of SADS-CoV N is essential for its interaction with RIG-I, and the regulation of SADS-CoV N binding to RIG-I is complex. At this stage, the detailed structural basis of this process is unknown, and the mechanism underlying modulation of the 2CARDs of RIG-I by SADS-CoV N also deserves further research. TRIM25-SADS-CoV N-RIG-I interaction supports the hypothesis that N protein may potentially impede the progression of TRIM25-RIG-I downstream signaling pathway. Different coronavirus N proteins have been indicated to inhibit IFN production. PDCoV N protein suppresses Riplet-induced ubiquitination of RIG-I through interaction with porcine RIG-I and TRAF3 protein [48]. SARS-CoV-1 and MHV N proteins interaction with PACT protein attenuates the activation of RIG-I and MDA5 [8]. PEDV N protein interferes with TBK1-mediated IRF3 phosphorylation and thus inhibits IFN-β expression [11]. Our current results indicated that ectopic expression of SADS-CoV N protein significantly inhibited mRNA expression of IFN-β, CXCL10 and ISG56 induced by SeV or poly I:C, and RIG-I activity. Overexpression of SADS-CoV N protein in HEK293T cells enhanced the replication of VSV-GFP (Figure 6). Thus, the SADS-CoV N protein facilitates viral replication by inhibiting the host interferon response.

In conclusion, our results suggested that TRIM25 impeded SADS-CoV replication. Furthermore, SADS-CoV N protein inhibits TRIM25 multimerization and TRIM25-RIG-I interaction to antagonize antiviral activity. In addition, SADS-CoV N suppressed IFN-β production and facilitated VSV-GFP replication. Our findings elucidate a significant molecular mechanism through which SADS-CoV utilizes its N protein to evade innate immune response mediated by TRIM25, thus providing an explanation of the natural viral defense mechanism and potentially facilitating the development of more effective strategies for controlling SADS-CoV infection.

### Supplementary Information

**Additional file 1. SADS-CoV N protein suppresses IFN-β production in IPI-2I cells.** **A**, **B** SADS-CoV N inhibits the transcription of IFN-β, CXCL10, and ISG56 after SeV infection and poly(I:C) transfection in IPI-2I cells. IPI-2I cells were transfected with empty vector or Myc-N for 36 h. Cells were stimulated with SeV infection (A) or poly(I:C) transfection (B) for 12 h. The relative mRNA levels of IFN-β, CXCL10, and ISG56 were evaluated by qRT-PCR. **C**, **D** SADS-CoV N inhibits phosphorylation of IRF3 and TBK1 stimulated by SeV and poly(I:C) in IPI-2I cells. IPI-2I cells were transfected with empty vector or Myc-N for 36 h. Cells were stimulated with SeV infection (C) or poly(I:C) transfection (D) for 12 h. The cell lysates were subjected to Western blotting analysis.

**Additional file 2. PCR primer sequences used in this study.**

**Additional file 3.** **qRT-PCR primer sequences used in this study.**

## Data Availability

The data that support the findings of this study are available from the authors on reasonable request.
