# Supplementary material for: Swine acute diarrhea syndrome coronavirus nucleocapsid protein antagonizes the IFN response through inhibiting TRIM25 oligomerization and functional activation of RIG-I/TRIM25
Source: Vet Res. 2024 Apr 8;55:44. doi: 10.1186/s13567-024-01303-z (PMC11000385; doi:10.1186/s13567-024-01303-z)
Supplement: Supplementary file 2 — Additional file 2. PCR primer sequences used in this study. [file 13567_2024_1303_MOESM2_ESM.docx]

**Additional file 2 PCR primer sequences used in this study**

| Names | Sequences (5’-3’) | |
| --- | --- | --- |
| HA-TRIM25 F | AGATTACGCTGAATTAATGGGCGGAACTGTGCCC |  |
| HA-TRIM25 R | GATCTGCTAGCTCGACTACCTGGTGGAGCAGATGGAGA |  |
| Flag-TRIM25 F | AGATTACGCTGAATTAATGGCGGAACTGTGCCC |  |
| Flag-TRIM25 R | GATCTGCTAGCTCGACTACCTGGTGGAGCAGATGGAGA |  |
| GFP-RING F | GGACTCAGATCTCGAATGGCAGAGCTGTGCCC |  |
| GFP-RING R | GATCCCGGGCCCGCGTTACCAGACGTCGGCGGGT |  |
| GFP-B-boxes F | GGACTCAGATCTCGAACGCCGCCCGCC |  |
| GFP-B-boxes R | GATCCCGGGCCCGCGTTAGGCCTCCAGGTCGGCG |  |
| GFP-CCD F | GGACTCAGATCTCGACTGGTGGAGCATAAGACCTGC |  |
| GFP-CCD R | GATCCCGGGCCCGCGTTAAGGTCTGGACTTGGCCAGGAAG |  |
| GFP-SPRY F | GGACTCAGATCTCGAGAGCTCCTGGAGTATTACATTAAAGTCATCC |  |
| GFP-SPRY R | GATCCCGGGCCCGCGTTACTTGGGGGAGCAGATGGAGAG |  |
| Myc-SADS-CoV N F | ATGGAGGCCCGAATTATGGCCACTGTTAATTGGGGTGACGC |  |
| Myc-SADS-CoV N R | GCCGCGGTACCTCGACTAATTAATAATCTCATCCACCATCTCAACCT |  |
| GFP-N2a F | TCTCGAGCTCAAGCTAGAAGTGCTTCACGTTCACAGTCT |  |
| GFP-N2a R | TAGATCCGGTGGATCAATGTCAACAGACTGTGACGGC |  |
| GFP-N2b F | GGACTCAGATCTCGAGTTGCTGCAGTTAAACAAGCTTTGG |  |
| GFP-N2b R | GATCCCGGGCCCGCGGACAGCTCTGCTTCTTGGTTTGG |  |
| GFP-N2c F | GGACTCAGATCTCGATCACCTGCACCTGCCC |  |
| GFP-N2c R | GATCCCGGGCCCGCGGCGAGGACCAAAGCATTTACG |  |
| Flag-RIG-I F | TGACGATGACAAGCTTATGACCACCGAGCAGCG |  |
| Flag-RIG-I R | CTCTAGAGTCGACTGTCATTTGGACATTTCTGCTGGATCAAATGG |  |
| GST-RIG-I 2CARD F | CTCCAAAAATCGATGGTATGACCACCGAGCAGCG |  |
| GST-RIG-I 2CARD R | GATCTGCTAGCTCGATTAAGATCTTCTGTTTCAACATCTTTTATACCTTT |  |
| Flag-RIG-I-IN F | TGACGATGACAAGCTTATGACCACCGAGCAGCG |  |
| Flag-RIG-I-IN R | CTCTAGAGTCGACTGTTATTTAAGATGATGTTCACATATAAGCAGTGAA |  |
| Flag-RIG-I-IC F | TGACGATGACAAGCTTCCAGAATGCCAGAATCTTAGTGAGAATTCA |  |
| Flag-RIG-I-IC R | CTCTAGAGTCGACTGTCATTTGGACATTTCTGCTGGATCAAATGG |  |
