# Supplementary material for: Swine acute diarrhea syndrome coronavirus nucleocapsid protein antagonizes the IFN response through inhibiting TRIM25 oligomerization and functional activation of RIG-I/TRIM25
Source: Vet Res. 2024 Apr 8;55:44. doi: 10.1186/s13567-024-01303-z (PMC11000385; doi:10.1186/s13567-024-01303-z)
Supplement: Supplementary file 3 — Additional file 3. qRT-PCR primer sequences used in this study. [file 13567_2024_1303_MOESM3_ESM.docx]

**Additional file 3 qRT-PCR primer sequences used in this study**

| Names | Sequences (5’-3’) | |
| --- | --- | --- |
| hIFN-β F | TGGGAGGCTTGAATACTGCCTCAA |  |
| hIFN-β R | TCCTTGGCCTTCAGGTAATGCAGA |  |
| hCXCL10 F | GTGGCATTCAAGGAGTACCTC |  |
| hCXCL10 R | TGATGGCCTTCGATTCTGGATT |  |
| hISG56 F | CATACATTTCCACTATGG |  |
| hISG56 R | TACTCCAGGGCTTCATTCA |  |
| hGAPDH F | TCATGACCACAGTCCATGCC |  |
| hGAPDH R | GGATGACCTTGCCCACAGCC |  |
| sIFN-β F | AGCACTGGCTGGAATGAAAC |  |
| sIFN-β R | TCCAGGATTGTCTCCAGGTC |  |
| sCXCL10 F | CCCACATGTTGAGATCATTGC |  |
| sCXCL10 R | CATCCTTATCAGTAGTGCCG |  |
| sISG56 F | CTGACTCACAGCAACCATG |  |
| sISG56 R | CTTTCAGGTGTTTCACATAGG |  |
| sGAPDH F | ACCTCCACTACATGGTCTACA |  |
| sGAPDH R | ATGACAAGCTTCCCGTTCTC |  |
| hTRIM25 F | GCAGGATGTGCGGATGACTG |  |
| hTRIM25 R | GCGTCCAAGAGAGCCTTCAT |  |
| sTRIM25 F | AGCACCGACCTGGAGAACAA |  |
| sTRIM25 R | CCTGCTGTTTAGCTCTCACG |  |
| SADS-CoV N F | CCCCTAAACCGGCTCGTAA |  |
| SADS-CoV N R | CAGAATTAGGAACACGCTTCCA |  |
|  |  |  |
